# Swedish student nurses’ perception of peer learning as an educational model during clinical practice in a hospital setting—an evaluation study

**DOI:** 10.1186/s12912-015-0098-2

**Published:** 2015-10-02

**Authors:** Marie Stenberg, Elisabeth Carlson

**Affiliations:** Department of Care Science; Faculty of Health and Society, Malmö University, Jan Waldenströms gata 25, SE 214 28 Malmö, Sweden

**Keywords:** Clinical education, Clinical practice, Peer learning, Student nurses

## Abstract

**Background:**

Peer learning, a collaborative learning model has no tradition in clinical education for undergraduate student nurses in Sweden, and little is reported of the student experience. An increasing number of students have led to a pressing need for preceptors and clinical placements thus, highlighting the need for a supportive educational model. The objectives for the current study were to explore how student nurses’ evaluated peer learning as an educational model during clinical practice in a hospital setting, and to compare perceptions among student nurses from year one and three.

**Methods:**

A questionnaire developed for the purpose of this study was developed and responded to by 62 (year one) and 73 (year three) student nurses. Data were collected between 2011 and 2013. The questionnaire contained six open- ended and eight closed questions on a four point Likert-scale. Written responses were analysed by content analysis and the closed questions by using descriptive statistics. Mann–Whitney U-test was used to examine differences in relation to students from year one and three.

**Results:**

The peer learning experience was evaluated in a positive way. Statistical significance differences were shown for two out of eight closed questions. The peer learning activities were evaluated as supportive and relevant for learning. Three categories emerged from the content analysis: “a feeling of safety”, “a sense of competition” and “the learning experience”.

**Conclusion:**

A feeling of safety seems to be connected to students’ perception of increased learning and independence. However, the sense of negative competition needs to be addressed when students are prepared for the teaching and learning activities in the peer learning model. Finally, what needs to be further investigated is what challenges and opportunities the peer learning model presents to preceptors.

## Background

Clinical practice is a vital part of nursing education, and highly valued by student nurses [1]. Learning in clinical practice has traditionally involved one student nurse being precepted by one registered nurse, where the preceptor has tried to mediate knowledge and skills to the student. Previous studies have shown that preceptorship is time consuming with no real reduction of clinical work for precepting nurses [2, 3]. From a student perspective, the clinical environment can be perceived as stressful and not welcoming [4, 5], especially in a hospital setting [6] therefore, the relationship between the student and the preceptor is fundamental to the learning process [7]. However, in Sweden, as well as internationally the increasing number of student nurses subsequently leads to a pressing need for additional preceptors and clinical placements [8]. This coincides with financial pressure on healthcare services and declining retention rates of registered nurses. To address these challenges, new educational models for learning in clinical practice need to be implemented and evaluated. One way forward might be peer learning, a collaborative educational model, where students from the same social grouping learn from and with each other [9, 10]. The peer learning model is based on the assumption that learning is constructed during social interaction in collaboration with significant others inspired by Piaget’s and Vygotsky’s theories [11]. The model is often referred to as the 2:1 clinical placement model, were two students are placed with one preceptor to overcome placement shortages [12]. Students are active and equal partners sharing learning activities and participating in discussions and feedback [13]. In nursing programs, the model is often referred to during clinical practice in hospital settings [14–16], and the most common model of peer learning is when a senior student acts as a tutor for a novice student [17]. However, it has been suggested that a more favourable approach is to combine students from the same year as this will allow students to alternate the roles of tutor and tutee, and motivates students to become more involved in their own learning [17].

The effects of peer learning have been described in systematic reviews [13, 18]. Positive effects such as increased cognitive skills, self-confidence, autonomy, clinical skills and reasoning are highlighted. Furthermore, and perhaps more difficult to evaluate in other educational models, are increased self-evaluating skills, collaborative- and leadership abilities [13], critical thinking [15], and the opportunity to share the experiences of a fellow student without the immediate interference of the preceptor [19, 20]. However, some adverse effects have also been described, including students not being compatible with each other, not wanting to compete for clinical tasks, having to share the preceptor’s attention and time, and having less time to practice independently [13].

Clinical placements in hospital settings are learning environments that include several categories of staff, constant shift changes and patients with complex medical- and nursing needs. In this demanding environment, student nurses are supposed to link theoretical knowledge to practical skills, and to demonstrate initiative, independence and work management skills. On the other hand, preceptors are expected to balance complex patient care with precepting of high quality [2]. This highlights the need for a supportive educational model in hospital settings, supporting students and preceptors as well as being cost beneficial. Peer learning has no tradition in clinical education for undergraduate student nurses in Sweden and little is reported of the student experience. The objective of this study was to explore how student nurses’ evaluated peer learning as an educational model during clinical practice in a hospital setting. Furthermore we wanted to investigate whether nurse students perceived peer learning differently with regards to being a novice student in year one or being at the threshold of graduation in year three.

## Methods

For this evolutional study, data were collected by using a questionnaire.

### Setting and the peer learning model

Peer learning was implemented in 2011 as a collaborative project between three hospitals and the affiliated university in southern Sweden as a potential solution to the lack of preceptors at the hospitals. The peer learning model was implemented at medical and surgical wards according to the 2:1 model [12]. In our model, students from the same educational level were paired together by the clinical teachers, based on the criteria that they had a clinical placement at a ward that was part of the project. Year one students conducted clinical practice for 5 weeks and year three for 8 weeks. Each pair of students was supervised by a registered nurse designated as a preceptor supporting the students in the learning process and ensuring patient safety. As opposed to the traditional “one student to one preceptor model” where preceptors work together with the student; preceptors in the peer learning model are supposed to, first facilitate the collaboration between students, and second, be a sounding board for the students if any concerns arise in relation to patient care.

Structured learning activities were developed for year one (Table 1) and three (Table 2) respectively, focusing for example fundamental nursing care, medical technical skills and ethical issues. The learning activities were developed by the preceptors in collaboration with clinical teachers from the affiliated university. National professional guidelines and the learning outcomes set by the university guided the content of the learning activities, and were based on the collaboration between the peer. The students were supposed to identify what theoretical and practical knowledge they needed, without immediate interference of the preceptor.

**Table 1:** Example of a structured activity for year one students

Catheterization of the urinary bladder	
• What are the indications/contraindications for catheterization?	
• Where can you find reliable information and instructions on the practices?	
• What preparations do you have to consider?	
• What information and instruction should you give to the patient?	
• What and where do you document?	
• What risks are connected to indwelling catheter?	
• Describe and motivate what observations you need to obtain.	
• Reflect and discuss ethical dilemmas related to catheterization of the bladder.	
• Reflect and discuss alternative treatments.

**Table 2:** Example of a structured activity for year three students

Perform a risk assessment for one of the patients that you care for	
• Identify a risk/s to patient safety	
• Reflect on and motivate why the patient is exposed to the identified risk/s	
• Identify and assess the evidence base for a suitable risk assessment tool	
• Explain how you use the instrument and reflect on how you will involve the unique patient in the process	
• Conduct a nursing intervention plan and reflect on who else in the healthcare team you need to involve in the process	
• Propose and argue for how you, in a systematic way, follow up the result of the planned interventions	

### Participants

Invited to participate was a purposive sampling of 180 student nurses from year one (*n* = 76) and three (*n* = 104) who had participated in the peer learning project.

### Data collection

Data were collected between 2011 and 2013. At the end of the clinical placement students were asked to complete a questionnaire evaluating their peer learning experience. The questionnaire was developed for the purpose of this study in collaboration between nurse academics and clinical teachers. The questionnaire contained five open ended questions (Table 3), and eight closed questions on a four point Likert-scale ranging from: *not at all (1), to some extent (2), to a high degree (3), to a very high degree (4).* Added to the questionnaire were background variables for age, sex and year. The questionnaire was tested for face validity by four first and four third year student nurses to determine whether the questions were clear and understandable. We calculated Cronbach’s alpha for internal consistency and the value was found to be 0,879, indicating that the items are adequately inter-related [21].

**Table 3:** Presentation of the open-ended questions

• What were the best aspects of peer learning?	
• What were the worst aspects of peer learning?	
• What improvements could be made to the peer learning activities?	
• Are there any other areas that would be suitable for peer learning activities?	
• Any other comments?	

### Ethical considerations

The project was approved by the local ethical committee at the Faculty of Health and Society at Malmö University. It was stressed that completion of the questionnaire was voluntarily and non-participation would not affect the individual student nurse in any sense. Participants were assured of confidentiality. The questionnaires were handed out to all students on the last day of the clinical placement after the completion of assessment and grading. By completing and returning the questionnaire students gave their informed consent.

### Data analysis

Statistical analysis of the closed questions was performed by the Statistical Package for Social Sciences (SPSS) version 20. Data were analyzed by using descriptive statistics (mean and median). The Mann–Whitney U test was used to examine differences in relation to student nurses from year one and year three. The statistical significance level was set to 5 % (*p* = 0.05). The Mann–Whitney U test is a nonparametric test used to examine differences between two independent groups when the dependent variable is either ordinal or continuous, but not normally distributed.

Data of the open ended questions were analyzed by using manifest content analysis to identify categories [22]. Content analysis uses a set of procedures to make valid interferences from a text [23]. The open ended questions were analyzed independently by the two authors to ensure reliability. The open coding used terms direct from the original statements and reflected the meaning units of the text. Meaning units dealing with the same content generated the initial categories. The pre-understanding of the first author consisted of her previous experience as a registered nurse, preceptor and clinical teacher. To enhance validity and limit the risk of predetermined interpretation, the second author, not having taken part in the project earlier, independently read and coded the data. These analyses were then compared and discussed in order to adjust the system of categories. This made it possible to determine if the first author’s pre-understanding could have affected the analysis. Initial categories were reduced into broader categories taking into account the research question and the pre-understanding. The text was re-read by the first author to ensure that the categories covered all aspects of the data and adjustments were made. The analysis resulted in three categories: “*A feeling of safety”, “The learning experience” and “A sense of competition”.*

## Results

The study group consisted of 135 students, the majority were female (*n* = 107). The percentage of male students (18.5 %) is slightly higher than the ratio of Swedish male registered nurses. The participants were between 18 and 50 years old (mean 26). Sixty-two students were in their first year and 73 in their third year (Table 4).

**Table 4:** Demographic characteristics of students responding to the peer learning evaluation form

Variables	Total	Students in year 1	Students in year 3
	*n* = 135	*n* = 62	*n* = 73
Gender			
Female	107(79.3 %)	47(75.8 %)	60(82.2 %)
Male	25(18.5 %)	15(24.2 %)	10(13.7 %)
Missing	3(2.2 %)	0	3(4.1 %)
Age (Years)			
Mean	25	24	27
Range	18–50	18–50	20–46

Overall, the student nurses were positive of the peer learning experience. The highest mean values were computed for the questions *Were the peer learning activities relevant for your coming profession as a registered nurse* (3.40) and *Was your preceptor properly prepared for the peer learning activities* (3.37). The lowest score (2.77) was measured for the question *Were you properly prepared for your teaching function.*

We examined if any statistical differences existed between the two sub-groups, namely students in year one and year three. Out of the eight questions two were statistically significant (*p* ≤ 0.05). The questions *To what extent did you learn during the peer learning activities* and *Were the peer learning activities relevant for your coming profession as a registered nurse* proved to be statistically significant with more positive evaluations for students nurses in year one (Table 5).

**Table 5:** Item statistics and comparison of means for students in year one and three

	Total population (*n* = 135)		Year one (*n* = 62)	Year three (*n* = 73)	*p*-value^a^
	Mean (SD)	Median^b^	Mean (SD)	Mean (SD)	
To what extent did you learn during the peer learning activities?	3.19 (0.719)	to a high degree	3.33 (0.351)	3.07 (0.757)	*0.050*
To what extent did you gain new theoretical knowledge?	2.95 (0.829)	to a high degree	3.03 (0.802)	2.88 (0.849)	0.237
To what extent did you gain new practical skills?	3.10 (0.843)	to a high degree	3.18 (0.806)	3.04 (0.873)	0.363
To what extent did you think theory and practice have been merged during peer learning activities?	3.13 (0.733)	to a high degree	3.21 (0.661)	3.06 (0.785)	0.264
Were you properly prepared for your teaching function?	2.77 (0.818)	to a high degree	2.70 (0.760)	2.82 (0.867)	0.329
To what extent do you think that the peer learning activities are complements to traditional precepting?	3.14 (0.848)	to a high degree	3.18 (0.742)	3.11 (0.934)	0.994
Was your preceptor/s properly prepared for the peer learning activities?	3.37 (0.773)	to a very high degree	3.41 (0.739)	3.33 (0.805)	0.654
Were the peer learning activities relevant for your coming profession as a nurse?	3.40 (0.765)	to a very high degree	3.60 (0.557)	3.23 (0.874)	*0.017*

^a^Mann–Whitney U-test, significance level 5 %, significant levels in italics

^b^1 = not at all 2 = to some extent 3 = to a high degree 4 = to a very high degree

The categories that emerged from the open-ended questions of the student evaluations were: *A feeling of safety, The learning experience* and finally *A sense of competition*. Quotations are presented below in an attempt to illustrate students’ experiences.

### A feeling of safety

The students stated that the main advantage of the peer learning model was that working together and supporting each other decreased the anxiety when entering a new clinical environment. Students felt safer and less nervous when introduced to the staff and when facing new clinical challenges. The possibility to be able to ask any kind of questions, reflect and discuss with each other, without having to ask the preceptor first, contributed to students’ feeling of safety. The students indicated that it was less stressful to ask “stupid questions” as they shared a mutual understanding of being a student in clinical situations.

*“Safety is to be two in a new place, it is easier to ask about things, pushing and supporting each other”* (Year 3 student).

When the preceptor was not physically present it was perceived as a safe haven to have a fellow student to discuss with. The feelings described were that having someone next to you never allowed you to feel abandoned in any clinical situation.

### The learning experience

The category reflects various dimensions of students’ responses, and describes opportunities as well as obstacles for learning. Student experienced a sense of increased learning as they felt more responsible when they took turns teaching each other. In other words, students indicated that they had to assure that their knowledge and skills were sufficient and up to date.

*“Peer learning was very beneficial to my learning. I could discuss my nursing practice with my peer and study and research with her also. We learnt from each other’s knowledge in a positive way”* (Year 3 student).

Student came to realize that learning together was more rewarding than they had initially thought prior to participate in the peer learning model. The discussions and reflections between the students and the exchange and replenishment of knowledge were defined as ingredients that increased the learning experience. However, it was also mentioned that the learning situations could be stressful when a student felt less knowledgeable than the peer.

*“I felt the pressure to be as good as or even better than my fellow student as we are compared all the time.”* (Year 1 student).

### A sense of competition

A major concern for the students, and mainly described as an obstacle for learning, was how students felt that they competed for the preceptor’s attention, focus and time. Competition also occurred in relation to patient care situations. First year students mentioned competition connected to practice medical-technical skills, for example taking blood samples and performing cannulation.

*“/ / ....... that you have to share activities such as peripheral vein catheterization”* (Year 1 student).

The year three students focused more on the last part of their clinical practice when they are supposed to, independently, care for the whole patient group and the activities associated with direct patient care.

*“Sometimes you stand in each other’s way, both myself and my peer want to show the preceptor that we have the necessary knowledge and skills, then it becomes competitive”* (Year 3 student).

Competition was also connected with opportunities to practice their leadership abilities including organizing patient care, collaborating with medical and healthcare staff and time management. However, competition could also be something positive linked to students ‘triggering’ each other’s performance as they constantly compared themselves with each other. This resulted in that the individual student identified personal learning needs. They also took the necessary actions required to reach the intended learning outcomes.

## Discussion

Although the majority of students seemed to be satisfied with the peer learning experience there is a tendency that year one students are slightly more positive than the final year students. The questions *To what extent did you learn during the peer learning activities* and *Were the peer learning activities relevant for your coming profession as a registered nurse* were statistically significant, displaying more positive value for year one students. This can probably be explained by the fact that as a novice, more structured activities are needed when developing nursing skills [24]. We suggest that this can be contributed to how the structured learning activities, used in the peer learning model, realistically mirror the content comprising the nursing profession. It is also possible that the structure activities provide a safe learning environment when students know what is expected of them. The thematic analysis illustrates that the peer learning model strongly contributes to students’ feelings of being safe in a clinical environment. A safe clinical learning environment has been described in previous studies as “to ask anything” culture [25], “decrease in anxiety” [19], and “feeling comfortable in the learning relationship” [14]. We argue that a safe clinical learning environment made possible by peer learning, most likely contributes to increased learning, in line with Roberts [25] who recommended friendship as an important factor enhancing learning. Students learn a great deal by explaining their ideas to others and by participating in activities in which they can learn from their peers [10, 18]. This is explained in the present study by students perceiving a high degree of self-confidence, independence and increased learning by participating in the peer learning model. As suggested elsewhere [26, 27], and supported by the current findings, clinical education programs need to manifest the positive consequences of peer learning and integrate the model in clinical education.

However, even though the peer learning model seems to be beneficial to the students’ learning experience in clinical practice, some negative aspects were presented and need to be acknowledged. These aspects were related to students not feeling properly prepared to teach a fellow students as well as the presence of competition. In the current study, the students described how the initial feeling of safety sometimes turned into frustration when students competed for the preceptor’s attention, opportunities to perform nursing interventions or when student realized that they had different learning styles. The issues of competition and non-compatible students have been identified as major concerns for nurse educators in previous studies [13, 15, 18]. To avoid competition, some students in our study, suggested that they should be allowed to choose a friend as their peer, and not be randomly paired. However, as presented earlier this was not an option for the students in the present study, the reason being that the ability to collaborate in a health care team is fundamental to provide high quality care. Therefore, students need to practice collaboration based on a professional relationship rather than on friendship. What seems to be missing though, are educational strategies directed towards the negative aspects of competition. Therefore, we suggest that students are theoretically introduced to peer learning as an educational model prior to clinical practice. The introduction should include a presentation of the structured activities and how they can support the learning experience, as well as being supportive for the teaching and learning sessions between the peer. Furthermore, the aspect of competition needs to be acknowledged, and openly discussed to prepare the students, should this issue arise during collaboration. One needs to bear in mind though, that competition can be favourably for learning as students from the same year can compare their knowledge and skills. The comparison between the peers can act as a trigger that further students’ motivation to learn.

## Limitations

We acknowledge the fact that the results are interpreted in the light of the limitations that might occur in connection with a limited convenience sample in a single setting as well as the nature of self-reporting on variables and are therefore not generalizable. No general conclusions can be drawn as the evaluation only measures students’ reaction on the teaching methods according to Kirkpatrick [28].

## Conclusions

The peer learning model seems to have the potential to be a sound educational model complementing the more traditional ways of supervision in clinical practice, for example, the one student-one preceptor model. A feeling of safety seemed to be connected to the increased learning experience as it supported the students’ perception of independence. On the other hand, the sense of negative competition needs to be addressed when students are prepared for the teaching and learning activities in the peer learning model. The introduction should include a presentation of the structured activities and how they can support the learning experience as well as being supportive for the teaching and learning sessions between the peer. However, what need to be further investigated are what challenges and opportunities the peer learning model presents to preceptors, therefore, to be able to understand the full implications of peer learning we propose further studies exploring the experiences of preceptors.

## Abbreviations

SPSS: Statistical package for social sciences
MS: Marie Stenberg
EC: Elisabeth Carlson
